# Aortoesophageal Fistula

**DOI:** 10.5811/cpcem.2017.2.33141

**Published:** 2017-07-06

**Authors:** Ryan Roten, Ryan Peterfy

**Affiliations:** Desert Regional Medical Center, Department of Emergency Medicine, Palm Springs, California

## CASE PRESENTATION

**Image 1:**
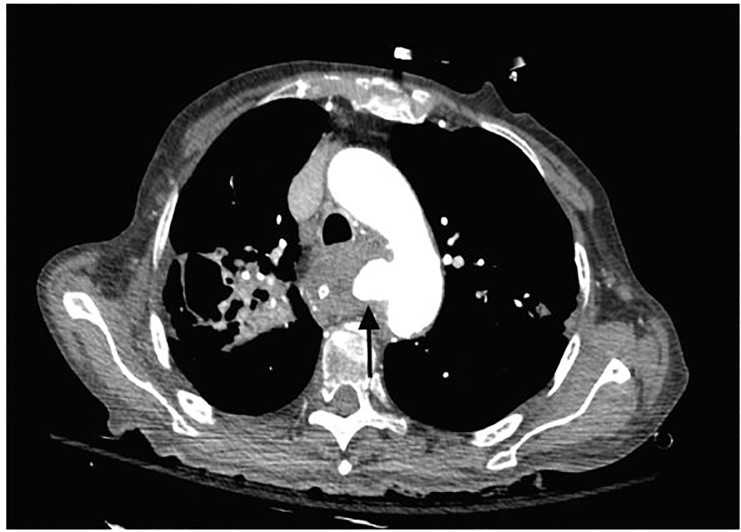
Computed tomography of the chest with contrast-filled aortoesophageal fistula (arrow).

A 90-year-old female presented after sudden collapse with a Glasgow Coma Score of 3, and profound hypotension. Shortly after endotracheal intubation, the patient developed significant hematemesis, and massive transfusion protocol was subsequently instituted. Computed tomography angiogram of the chest revealed active bleeding from an aortoesophageal fistula (Image 1). During the resuscitation, over four liters of blood were collected via oral gastric tube and manual suctioning by nursing staff before the resuscitation was terminated at the family’s request.

## DISCUSSION

The most common causes of aortoesophageal fistulas are thoracic aortic aneurysm, foreign body ingestion, postoperative complications, and esophageal malignancy. The classic presentation of mid-thoracic chest pain and sentinel arterial hemorrhage followed by exsanguination is known as Chiari’s triad.^1^ If the abnormality is identified early during the asymptomatic period using endoscopy or computed tomography angiogram of the chest, survival is possible with immediate surgical intervention or endovascular stenting.^2^ Medical providers must be familiar with the presentation, diagnostics, rapid interruption of diagnostics and treatment of aortoesophageal fistulas to make survival of this rare and typically fatal pathology possible.

CPC-EM Capsule

What do we already know about this clinical entity?

Aortoesophageal fistula is a rare and typically fatal pathology that presents as massive upper gastrointestinal hemorrhage.

What is the major impact of the image(s)?

This is a rarely seen image, given the degree of extremis and high mortality of patients with aortoesophageal fistula at emergency department presentation.

How might this improve emergency medicine practice?

Emergency medicine providers must be familiar with the presentation, diagnostics, rapid interruption of diagnostics and treatment of aortoesophageal fistulas.

## Figures and Tables

**Image 2:**
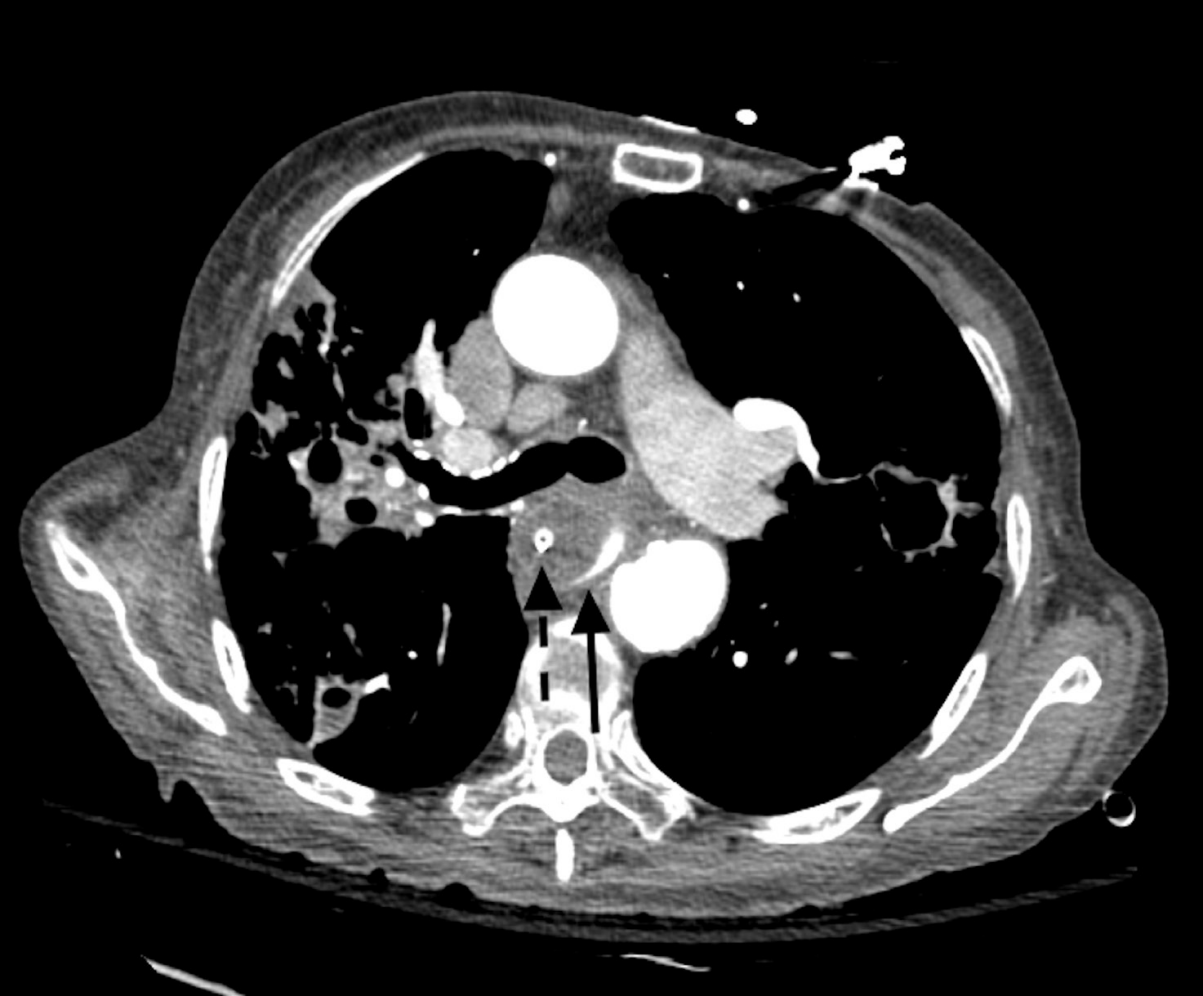
Computed tomography of the chest with contrast filling the esophagus (solid arrow) and adjacent oral gastric tube (dashed arrow).
